# Supplementary material for: Corin Deficiency Alters Adipose Tissue Phenotype and Impairs Thermogenesis in Mice
Source: Biology (Basel). 2022 Jul 23;11(8):1101. doi: 10.3390/biology11081101 (PMC9329919; doi:10.3390/biology11081101)
Supplement: Supplementary file 1 [file biology-11-01101-s001.zip › biology-1804939-supplementary.pdf]

Corin Deficiency Alters Adipose Tissue Phenotype and Impairs Thermogenesis in Mice

Supplementary Figures and Table

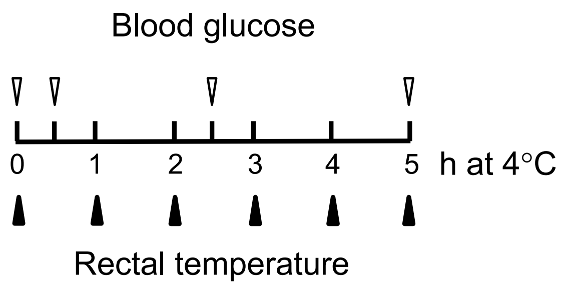

Supplementary Figure S1. Schematic diagram of the cold exposure experiment. WT and corin KO mice (4-month-old males) (*n* = 9-13) were kept at 4°C for 5 h. Time points, at which blood glucose levels (open arrowheads) and rectal temperatures (black arrowheads) were measured, are indicated.

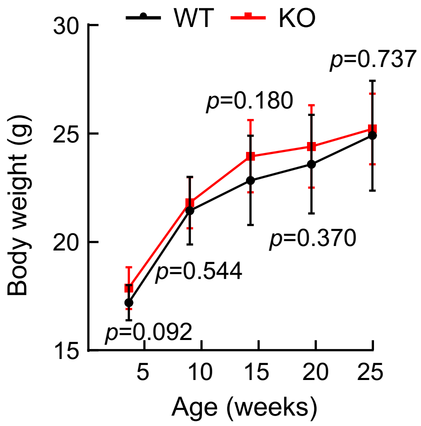

Supplementary Figure S2. Body weights in female WT and corin KO mice. Body weights in female WT and corin KO mice on chow diet were measured between 5 and 25 weeks of age (n = 11). Data of mean ± SD are presented. *P* values between the two groups at the same age were assessed by Student's *t* text.

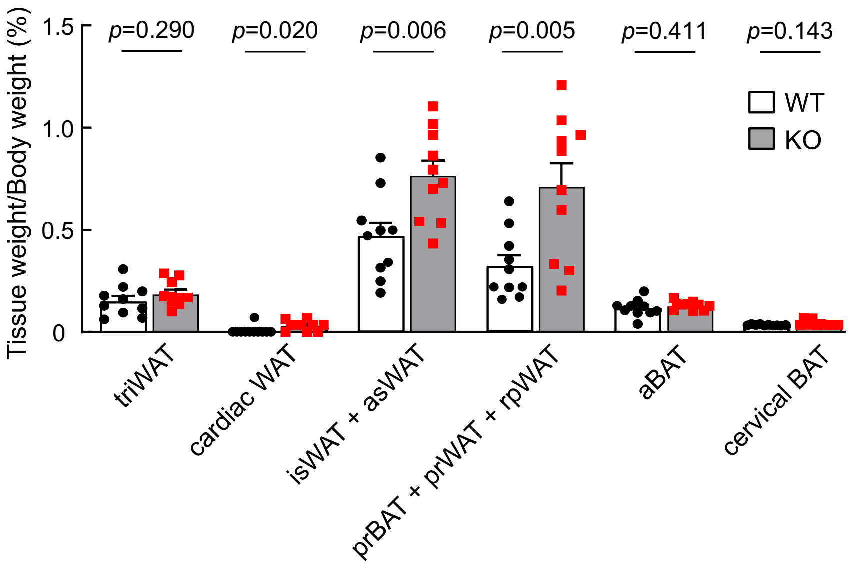

Supplementary Figure S3. Data of additional adipose tissue weights in WT and corin KO mice**.** Ratios of adipose mass *vs*. body weight were analyzed in WT and corin KO mice (males, 4 months old) (n = 10). triWAT: triceps-associated WAT; isWAT: interscapular WAT; asWAT: anterior subcutaneous WAT; prWAT: perirenal WAT; rpWAT: retroperitoneal WAT; prBAT: perirenal BAT; and aBAT: axillary BAT. Data of mean ± SEM are shown. Due to difficulties in cleanly dissecting minor adipose tissues, grouped isWAT and asWAT (isWAT+asWAT) and prBAT, prWAT, and rpWAT (prBAT+prWAT+rpWAT) weight data are shown. *P* values were analyzed by Student's *t* test (triWAT, isWAT + asWAT, prWAT + rpWAT + prBAT, and aBAT) or Mann-Whitney test (cardiac WAT and cervical BAT).

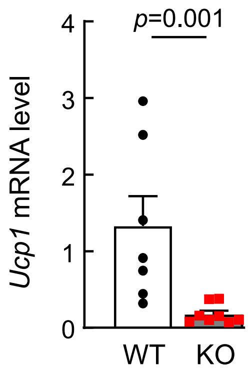

Supplementary Figure S4. *Ucp1* mRNA levels in ingWAT from WT and corin KO mice. *Ucp1* mRNA levels in ingWAT from 4-month-old male WT and corin KO mice were examined by qRT-PCR (*n* = 7-8). Data of mean ± SEM are presented. *P* value was examined by Mann-Whitney test**.**

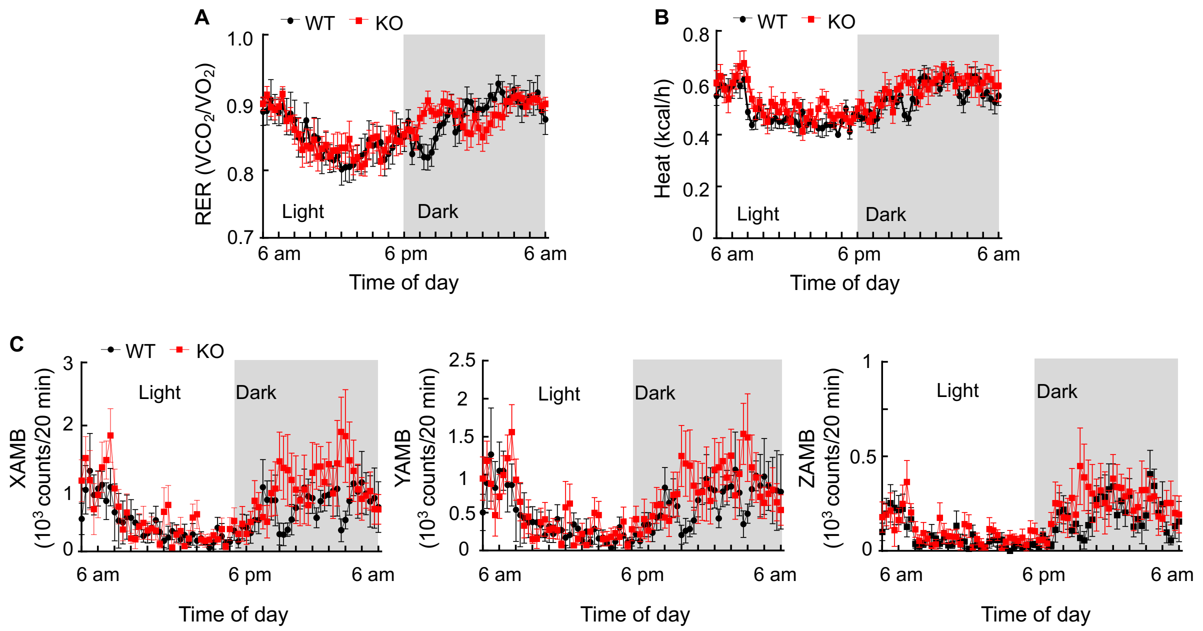

Supplementary Figure S5. Additional metabolic data from WT and corin KO mice. Respiratory exchange ratio **(**RER) (**A**), heat production (**B**), and motor activities (**C**) in WT and corin KO mice (4-5-month-old males) (n = 8) were monitored in 12-h light and dark cycles. In (**C**), ambulatory movements in X-axis (XAMB), Y-axis (YAMB), and Z-axis (ZAMB) are shown. Data of mean ± SEM are presented. P values were examined by Student's t test.

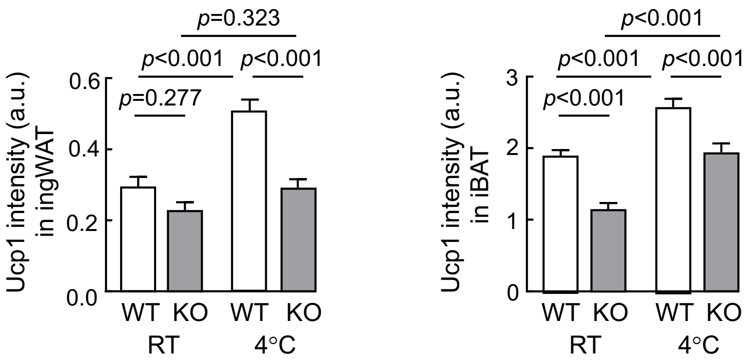

Supplementary Figure S6. Ucp1 staining intensities in ingWAT and iBAT from WT and corin KO mice. Immunostaining of Ucp1 protein was done in ingWAT (left panel) and iBAT (right panel) sections from WT and corin KO mice at RT or 4°C. Relative Ucp1 staining intensities in arbitrary units (a.u.) were examined using Image Pro software. At least 4 fields from each section and at least 4 sections per mouse (n = 3 per group) were examined. Each group includes at least 48 data points. Individual dots are not shown. Data of mean ± SEM are shown. P values were analyzed by two-way ANOVA and Tukey *post hoc* analysis.

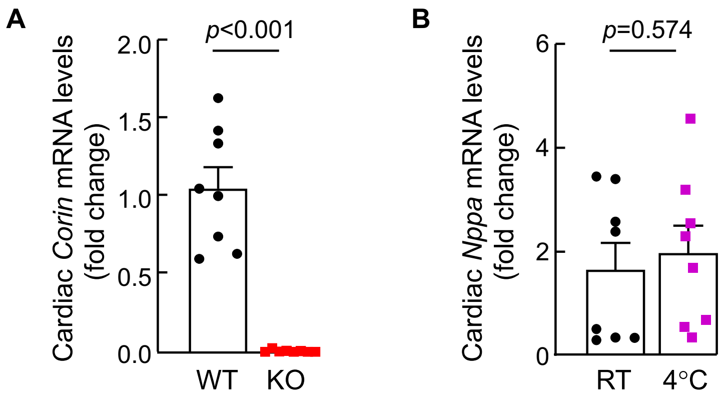

Supplementary Figure S7. Cardiac *Corin* and *Nppa* mRNA expression in corin KO mice. Cardiac *Corin* (**A**) and *Nppa* (**B**) mRNA levels in corin KO mice (males, 4 months old) at RT and 4°C were analyzed with qRT-PCR (n = 8). Data presented are mean ± SEM. *P* values were analyzed by Mann-Whitney test.

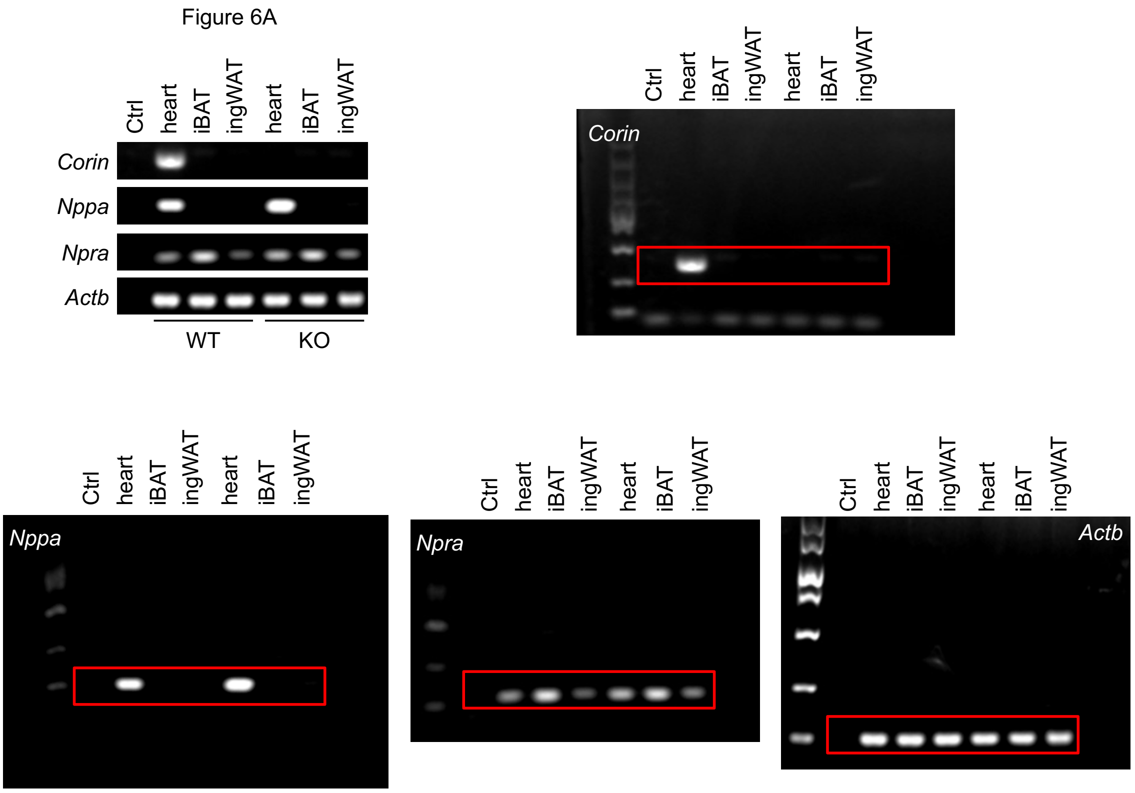

Supplementary Figure S8. Uncropped gel images of Figure 6A.

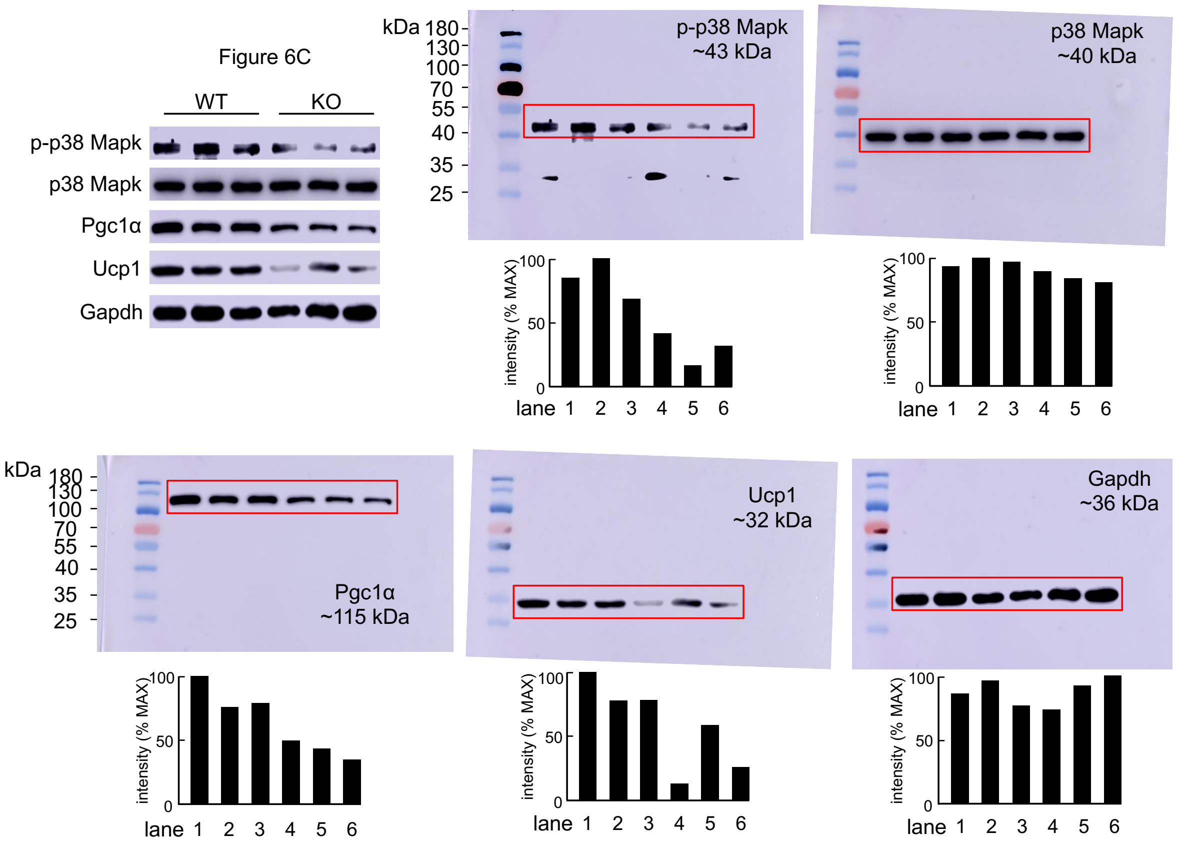

Supplementary Figure S9. Uncropped western blot images and relevant intensities of bands in Figure 6C.

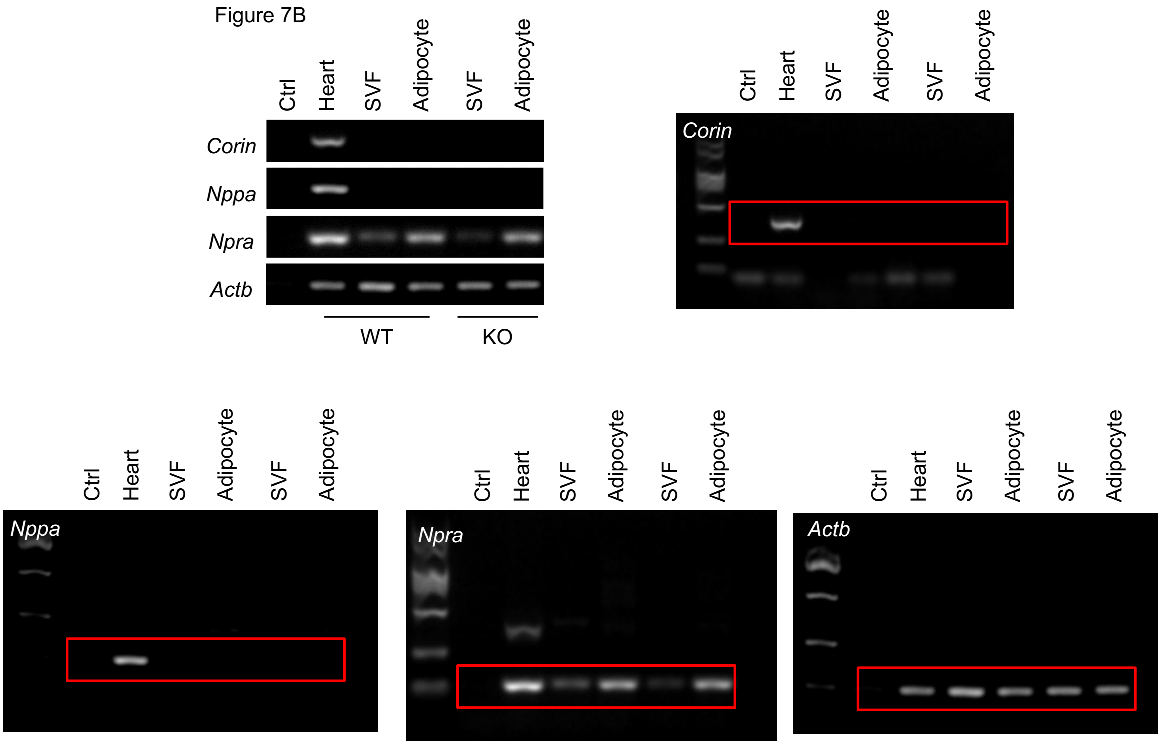

Supplementary Figure S10. Uncropped gel images of Figure 7B.

Supplementary Table S1. Primers used in qRT-PCR

Gene	Locus	Sequence	Size (bp)
Corin F	NM_001122756	5'-ATCACTCACAGCCAGTGTCAA-3'	390
Corin R		5'-TCCACAGAGTGATTGCTTTCCAT-3'	
Nppa F	NM_008725.3	5'-TCGTCTTGGCCTTTTGGCT-3'	106
Nppa R		5'-TCCAGGTGGTCTAGCAGGTTCT-3'	
Pgc1α F	NM_008904.2	5'-CCCTGCCATTGTTAAGACC-3'	158
Pgc1α R		5'-TGCTGCTGTTCCTGTTTTC-3'	
Ucp1 F	NM_009463.3	5'-GGCCTCTACGACTCAGTCCA-3'	84
Ucp1 R		5'-TAAGCCGGCTGAGATCTTGT-3'	
Cidea F	NM_007702.2	5'-GCCGTGTTAAGGAATCTGCTG-3'	166
Cidea R		5'-TGCTCTTCTGTATCGCCCAGT-3'	
Cpt1b F	NM_009948.2	5'-TTCAACACTACACGCATCCC-3'	117
Cpt1b R		5'-GCCCTCATAGAGCCAGACC-3'	
Cpt2 F	NM_009949.2	5'-AGTATCTGCAGCACAGCATCGTA-3'	48
Cpt2 R		5'-GGCTTCTGTGCACTGAGGTATCT-3'	
Cox7a1 F	NM_009944.3	5'-AGAAAACCGTGTGGCAGAGA-3'	112
Cox7a1 R		5'-CAGCGTCATGGTCAGTCTGT-3'	
Actb F	NM_007393.5	5'-ACGGCCAGGTCATCACTATTG-3'	87
Actb R		5'-CACAGGATTCCATACCCAAGAAG-3'	
Hprt F	NM_013556.2	5'-TCATTATGCCGAGGATTTG-3'	100
Hprt R		5'-GCCTCCCATCTCCTTCAT-3'	
